# The physiological and molecular mechanism of brassinosteroid in response to stress: a review

**DOI:** 10.1186/s40659-018-0195-2

**Published:** 2018-11-12

**Authors:** Ali Anwar, Yumei Liu, Rongrong Dong, Longqiang Bai, Xianchang Yu, Yansu Li

**Affiliations:** 1 The Institute of Vegetables and Flowers, Chinese Academy of Agricultural Sciences, Beijing, 100081 China; 2 College of Agricultural and Biological Engineering, Heze University, Heze, 274015 China

**Keywords:** Brassinosteroids, Physiology, Antioxidants, Biotic and abiotic stress

## Abstract

The negative effects of environmental stresses, such as low temperature, high temperature, salinity, drought, heavy metal stress, and biotic stress significantly decrease crop productivity. Plant hormones are currently being used to induce stress tolerance in a variety of plants. Brassinosteroids (commonly known as BR) are a group of phytohormones that regulate a wide range of biological processes that lead to tolerance of various stresses in plants. BR stimulate BRASSINAZOLE RESISTANCE 1 (BZR1)/BRI1-EMS SUPPRESSOR 1 (BES1), transcription factors that activate thousands of BR-targeted genes. BR regulate antioxidant enzyme activities, chlorophyll contents, photosynthetic capacity, and carbohydrate metabolism to increase plant growth under stress. Mutants with BR defects have shortened root and shoot developments. Exogenous BR application increases the biosynthesis of endogenous hormones such as indole-3-acetic acid, abscisic acid, jasmonic acid, zeatin riboside, brassinosteroids (BR), and isopentenyl adenosine, and gibberellin (GA) and regulates signal transduction pathways to stimulate stress tolerance. This review will describe advancements in knowledge of BR and their roles in response to different stress conditions in plants.

## Introduction

**Fig. 1:**
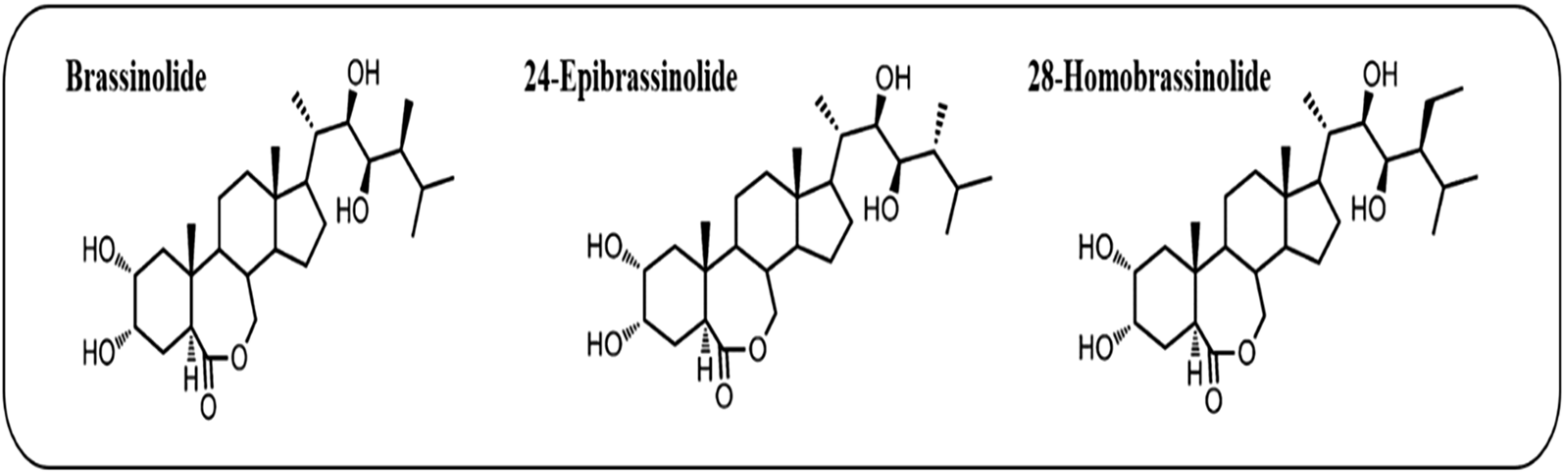
Structure of three common brassinosteroids

**Fig. 2:**
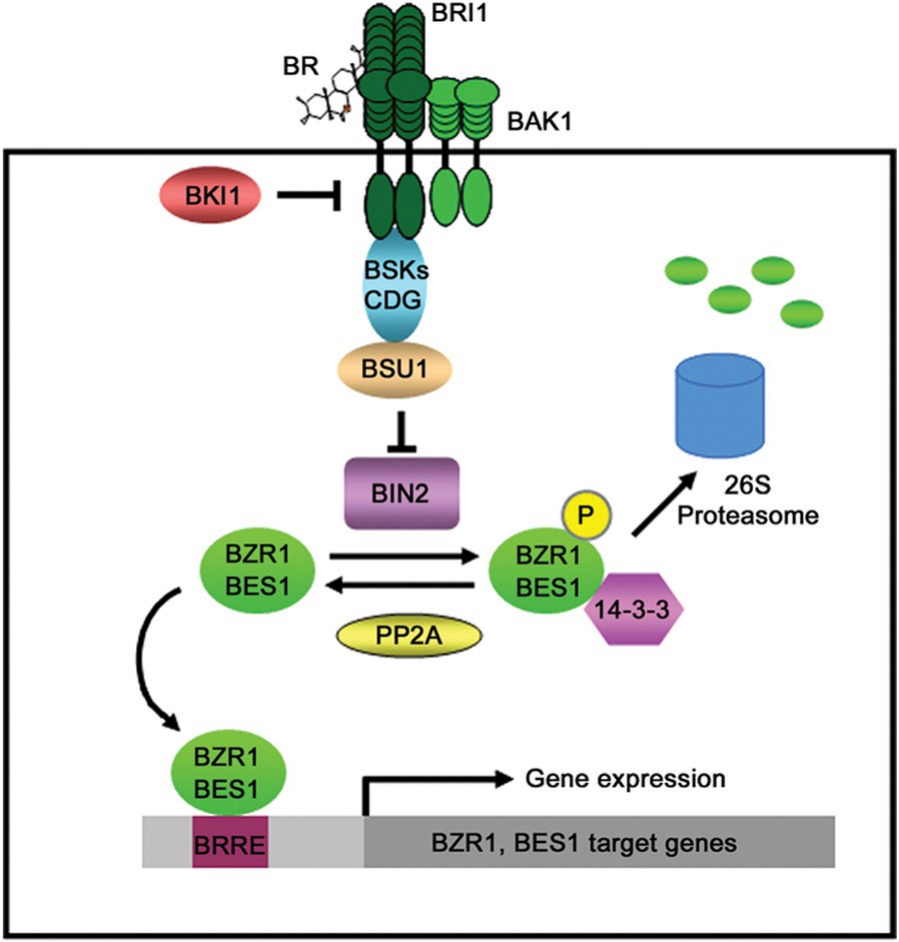
Model of brassinosteroid (BR) signaling in Arabidopsis, as reported in *Plant Signaling & Behavior* 2013 [93]

**Fig. 3:**
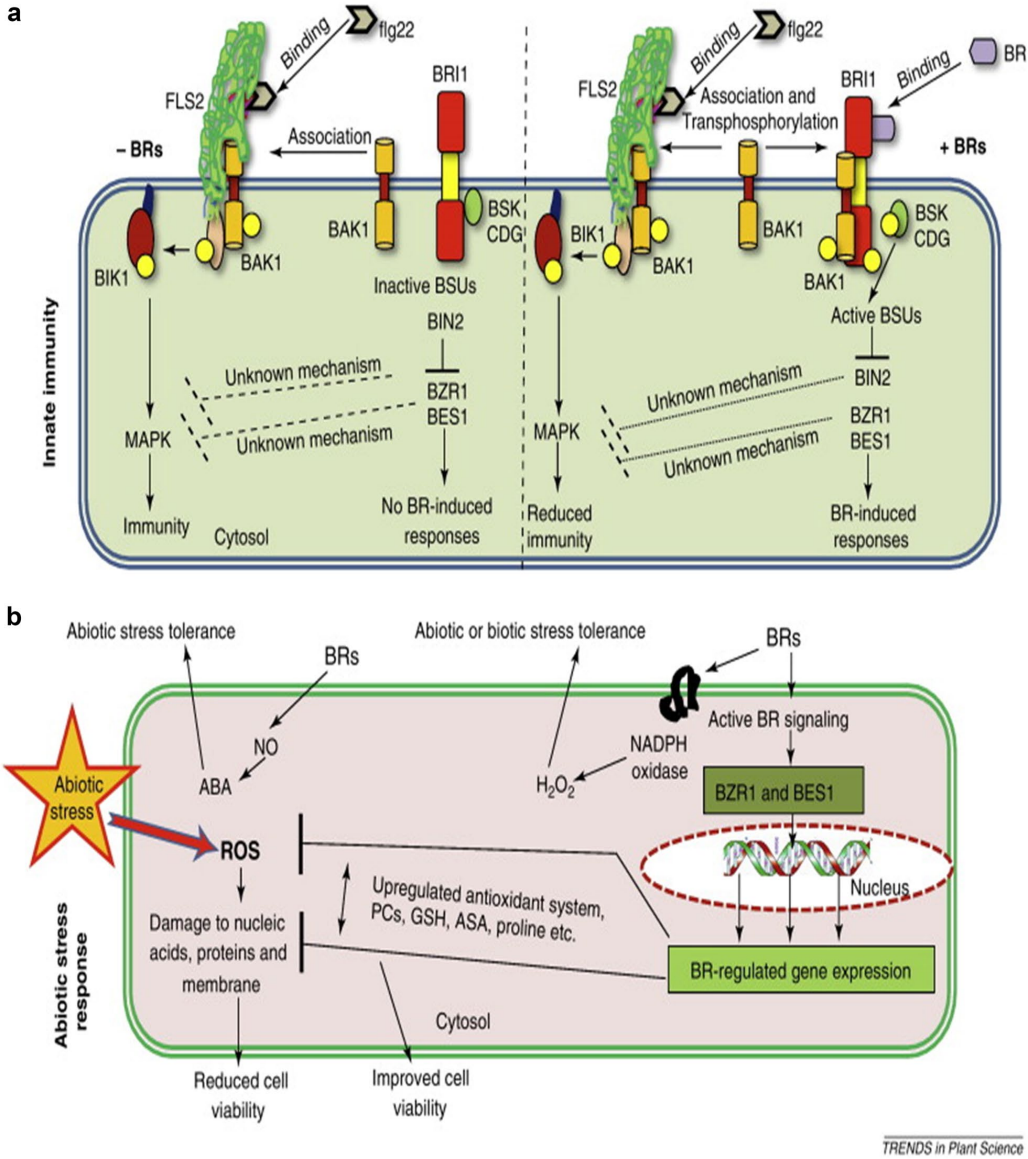
Model of brassinosteroids (BRs) implicated in innate immunity (**a**) and abiotic stress (**b**) responses in model plant (*Arabidopsis*), as reported in Trends in Plant Science 2012;17: 594–605. (10.1016/j.tplants.2012.05.012) [3]

Brassinosteroids (BR) are a group of plant steroid hormones that were first isolated from *Brassica* pollen about 40 years ago. Around 60 related compounds have been identified [1–3], however, brassinolide, 24-epibrassinolide, and 28-homobrassinolide are the most bioactive BR. These three BR (Fig. 1) are widely used in physiological and molecular studies [4]. BR are similar to animal steroid hormones that facilitate processes starting in embryonic development up to adult homeostasis [5], through a complex signal transduction pathway, presented in Fig. 2. Earlier studies revealed that BR regulate diverse physiological and developmental processes in plant diversity, such as cell elongation (stem, root), leaf expansion, photomorphogenesis, flower developmental processes, male sterility, stomatal developmental processes, and resistance to stress [1–3, 6–9]. Like their animal counterparts, BR upregulate thousands of genes (Fig. 3) [2] related to cell division and differentiation [5, 10], which leads to control over all developmental process [11].

24-Epibrassinolide is a biologically active BR compound [12] that plays a critical role in developmental processes [12–15] and regulates cell division and elongation [5], gene expression, and vascular differentiation [3]. BR regulates various physiological and developmental process; cell division and elongation, vascular differentiation, seed germination up to maturation [8, 9]. Moreover, BR increase resistance in plants to various kinds of abiotic stress (low and high temperature, drought, heat, salinity, Ca(NO_3_)_2_, and heavy metal toxicity) [2, 14–16]. BR improve the plant defense system to tolerate various stresses by increasing chlorophyll contents, which ultimately increases photosynthetic capacity, enhances antioxidant system capacity, increases enzymatic activity, and upregulates stress response genes [superoxide (SOD), peroxide (POD), catalase (CAT), glutathione reductase (GR) and ascorbate peroxide (APX)] [17, 18]. Exogenous BR also increased cucumber seedling growth under Ca(NO_3_)_2_ stress by enhancing photosynthetic capacity, the antioxidant system, and chloroplast ultra-structure [14]. BR applications under normal conditions also stimulate plant growth, net photosynthetic rate, and antioxidant system capacity, reflecting their dynamic roles. BR insensitive mutants were identified in model plants *Arabidopsis* and *Brassica* [19, 20], and the plants had many growth defects, like dwarfism, deep green leaves, late flowering, and male sterility [21–23]. These findings indicate that BR has a positive response to various kinds of stress and activates different physiological and molecular mechanisms to enhance plant growth to induce stress tolerance. This review briefly discusses the physiological and molecular mechanisms of BR under different stress conditions.

## Regulatory mechanisms of BR in plants

**Fig. 4:**
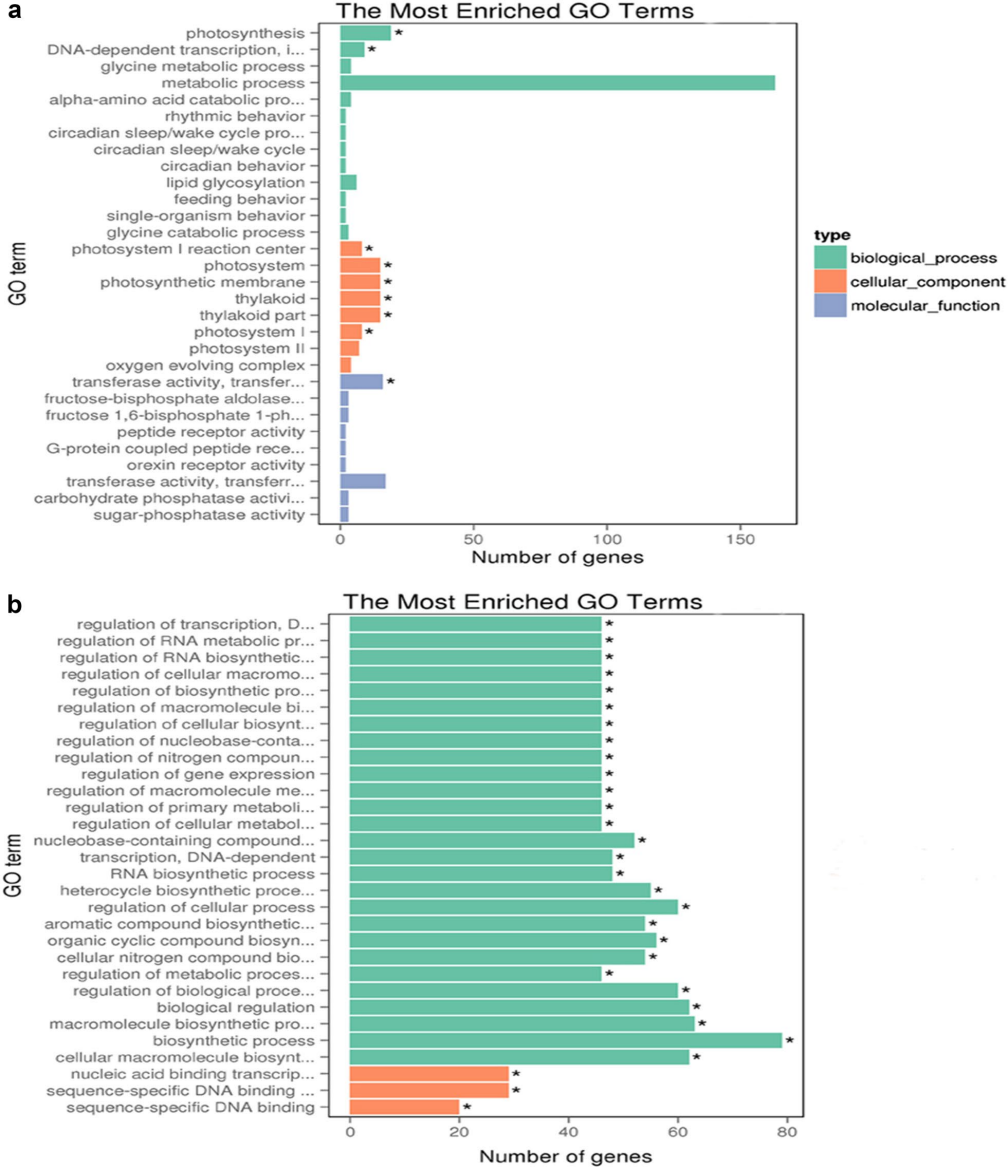
GO enrichment analysis of DEGs between Chill and Chill + BR treatment. **a** Up-regulation; **b** down-regulation, as reported in Front Plant Sci., 29 August 2016. 10.3389/fpls.2016.01281. [2]

The signal transduction pathway of BR has been intensively studied over the past decade (Fig. 2) and these studies have established a complex BR signal transduction pathway, that play an important role in plant growth and development. The signal transduction pathway shows that BR is perceived by BRASSINOSTERIOD INSENSITIVE 1 (BRI1) receptor kinase at the cell surface and activates BRASSINAZOLE RESISTANT 1 (BZR1) and BRI1-EMS SUPPRESSOR 1 (BES1) transcription factors to induce stress tolerance. Exogenously applied BR binds to BRI1 inducing an association with BRI1-ASSOCIATED RECEPTOR KINASE 1 (BAK1) and disassociation of BRI1 KINASE INHIBITOR 1 (BKI1) (Fig. 3). Sequential transphosphorylation between BRI1 and BAK1 is required to activate BRI1 and furthermore to phosphorylate BR-SIGNALING KINASE 1 (BSK1) and enhance BRI1 SUPPRESSOR 1 (BSU1) activity. The activated BSU1 inhibits BRASSINOSTEROID INSENSITIVE 2 (BIN2) through dephosphorylation of the phospho-tyrosine residue of BIN2, which allows accumulation of unphosphorylated BZR1 and BZR2/BES1 transcription factors. The dephosphorylated BZR1 and BES1 enter the nucleus and function in regulating BR-targeted genes to enhancing plant stress tolerance [4, 22, 24] by increasing the capacity of antioxidant enzymes [4], regulating accumulation of endogenous hormones [15, 25], and upregulating thousands of genes [2] (Fig. 4).

## Physiological mechanisms of BR in plants under stress conditions

### Seed germination and growth

Seed germination and seedling growth are important parameters for healthy crop production. Seeds are very delicate during germination and can be easily damaged by minor external stimuli (stress) [26]. BR enhancing germination rate and ultimately increase seedling growth under stress conditions [27–29]. BR control the key genes G-protein α-subunit 1 (GPA1) and G-protein coupled receptor 1 (GCR1) that are responsible for seed germination. GPA1 is a subunit of the heterotrimeric G protein and GCR1 is a putative G protein-coupled cell surface receptor [26]. *GPA1* (G-protein α-subunit 1) is a key gene that regulates various biological processes, including biotic and abiotic stresses, growth and developmental processes, biosynthesis of flavonoid, as well as activating transcription factors and nutrient transporters [23]. *GPA1* interacts with *Pirin1* and regulates early seed germination and seedling growth [26]. Previous studies have shown that BR significantly increased: tomato seedling growth under low temperature and weak light stress [17]; cucumber seedling growth under Ca(NO_3_)_2_ stress [18]; pepper shoot growth, fresh and dry weight under stress [11]. In the study on tomatoes the authors concluded that BR regulate plant antioxidant enzyme activities as well as increase nitrogen metabolism, chlorophyll, and accumulation of nutrients (Figs. 3, 4) [17]. BR-deficient *dwarf1* mutant in rice, which lacks activity of BR C-6-oxidase, exhibits abnormal organization and polar cell elongation of leaf, stem, and other developmental organs [30]. FERONIA (a receptor-like kinase required for normal pollen tube development and cell elongation) is involved in BR signaling and ethylene signaling to control hypocotyl elongation during seedling growth, indicating antagonistic relationship between BR and ethylene [31]. Stress causes membrane damage, reduces CO_2_ intake and as a result of stomatal closure, decreases hydrolytic enzyme activity and increases lipid peroxidation level. BR application regulates those activities [32] and maintains proper seedling growth under stress conditions [17]. Taken together, BR increases stress tolerance by enhancing the plant defense system including increasing the activities of antioxidant enzymes (APX, SOD, POD, CAT and GR) through increase the antioxidant genes expression, and altering nutrient accumulation to enhance seed germination and seedling growth.

### Shoot and root growth

BR application at very low concentrations significantly increases plant growth, while higher levels have adverse effects on growth and growth-related parameters at early stages [3]. Plants showed very rapid responses upon BR application [33] by increasing shoot and root growth [30]. Many previous studies have demonstrated that increased BR during plant growth is because of elongation and expansion of cells [8, 20, 30, 34]. Many BR loss-of-function mutants showed significant decrease in cell size, leaf epidermal and mesophyll cell number [30], whereas plants overexpressing the BR receptor *BRI1* showed significant increase in the size of epidermal and mesophyll cells [20]. Brz-insensitive-long hypocotyl 4 *(BIL4)* is a key activator of the BR signal pathway, which interacts with receiver BR receptor *BRI1* and plays a dynamic role in controlling cell elongation: *BIL4*-*OX* mutants had increased cell size and height, while these traits decreased in *BIL4*-*RNAi* mutants compared with wild type plants [20, 35].

*AtRALF* (Rapid alkalinization factor; is a peptide signal that play a key role in cell biology and mostly regulate cell expansion) mutants had significantly increased root cell size and down-regulate the transcription level genes involved in BR biosynthesis genes (*CPDs* and *DWFs*), upon exogenous BR application, compared with wild-type plants [5]. The authors suggested that the *RALF* signaling mechanism could be positively interfering with the BR signaling pathway, to regulate root cell growth and elongation. However, loss-of-function of *brl1* mutants caused abnormal phloem and xylem differentiation and also increased vascular defects [20], suggesting that BR receptors played a fundamental role in cell growth (roots and shoots) and vascular differentiation. Taken together the evidence illustrated that the BR signaling pathway played a fundamental role in plant cell differentiation and increased induced stress tolerance [21, 36–38].

### Leaves

Leaves are specialized organs, where photosynthesis, respiration, transpiration, and guttation take place. BR control a broad spectrum of physiological and developmental processes, including processes occurring in the leaves. In a recent study, the growth of leaves (leaf area and leaf mass per unit leaf area) was significantly increased in cucumber upon BR application [2]. Previous studies reported that BR application significantly increased leaf area, chlorophyll content, and photosynthesis, which are the key factors for plant growth under stress [14, 15]. In a recent study, the *super compact*-*2* mutant of cucumber was identified showing the typical BR biosynthesis-deficient phenotype. These mutants developed dark green and wrinkled leaves (reduced leaf area), defects in cell elongation and vascular development, and reduced endogenous BR accumulation [20, 21]. The authors concluded that *scp*-*2* is a BR biosynthesis-defective mutant, which is due to a mutation of *CsDET2* (*steroid 5*-*alpha*-*reductase DET2*), a gene that plays a key role in BR biosynthesis in cucumber [21]. These results stand out the active role of BR in leaf developmental process.

### Chlorophyll and photosynthesis

Chlorophyll and photosynthesis are essential for plant growth. Application of exogenous BR significantly increased chlorophyll content and enhanced photosynthetic characteristics of plants under various stresses [2, 39]. Chlorophyll is an important parameter frequently used as an indicator of chloroplast development and photosynthetic activity [17]. Chlorophyll is highly sensitive to external stimuli (stress) that decrease total chlorophyll a, b, and carotenoid contents in leaves [37]. BR protect chlorophyll from external stimuli and also increase their concentration under various stresses [3, 36, 38, 40–44]. Exogenous BR significantly promote chlorophyll and photosynthetic parameters under low temperature, weak light stress, and Ca(NO_3_)_2_ stress [17, 18]. Moreover, BR can regulate the combination of chlorophyll molecule (by regulating chlorophyllase activity) with membrane protein and maintain stability of the thylakoid membranes [38, 43]. Photosynthesis is the basis of plant growth and development, where plant convert light energy to chemical energy (carbohydrates, such as sugars) that used in various developmental activities [45, 46]. Chlorophyll is one of the basic units of photosynthesis and appropriate levels of chlorophyll are required for proper photosynthesis [3], however, chlorophyll is very sensitive to abiotic stress.

Brassinosteroids regulate and maintain photosynthetic activity under water stress conditions in tomato seedlings [17, 47]. BR alleviate the adverse effect of different stress conditions and regulate the defense system by regulating transcription levels of defense genes in cucumber [48]. Moreover, BR regulate the Rubisco activase (*RCA*) gene, which plays a key role in photosynthesis, under drought and heat stress in wheat. In addition, BR significantly increases the activities of antioxidant enzymes and the process of photosynthesis [45], also BR-treated seedlings increased in: CO_2_ assimilation and quantum yield of photosystem II (PSII); ribulose-1, 5-bisphosphate carboxylase/oxygenase (Rubisco) activities and expression of Rubisco large subunit (*rbcL*) and Rubisco small subunit (*rbcS*) genes to increase photosynthetic capacity [1]. These findings suggest the positive role of BR in photosynthesis [1]. Moreover, transcriptome analysis of BR shows that BR increases a variety of chlorophylls and photosynthesis-related genes under low temperature stress (Fig. 4) [2]. Thus, BR promote accumulation of chlorophylls and photosynthetic capacity by regulating a variety of enzymes including chlorophyllase and Rubisco, transcript levels of encoded genes involved chlorophyll and photosynthesis under stress [1, 2, 45, 46].

### Nutrient uptake and regulation

Uptake of essential nutrients (N, P, K, Ca, Mg, Fe, Mn, Cu, Zn etc.) is also very important for plant growth. Higher ion influx allows increased efficiency of light energy transformation, CO_2_ conductivity, potential of light and dark reactions, and photosynthetic rate [13, 49]. A recent study reported that BR increased essential inorganic ions, decreased toxic ions, and promoted ion homeostasis especially in leaves, root, and epicotyl of canola under salt stress [12]. Reducing the harmful effect of low temperature and weak light stress, 24-epibrassinolide enhanced nitrogen metabolism; the activity of nitrate reductase (NR), nitrite reductase (NiR), glutamine synthetase (GS), glutamate synthase (GOGAT) and glutamate dehydrogenase (GDH) enzymes, and induced photosynthetic characteristics of tomato seedlings [17]. Furthermore, exogenous BR application increased H^+^-ATPase and Ca^2+^-ATPase activities in root and leaf [50], which are responsible for establishing an electrochemical potential gradient to maintain ion balance in plants to alleviate stress effect.

### BR interaction with endogenous plant hormones

Plant hormones (ethylene, ABA, IAA, ZR JA, auxin, GA) are the basic units of plant growth and development and they promote stress tolerance [51]. The regulation of endogenous hormones is also very important for enhancing stress resistance [51, 52]. Therefore, crosstalk and interaction of BR with plant hormones are very essential for growth and stress responses [50, 51, 53–55]. Previous studies reported that ABA acts as a signal molecule in response to stressed and unstressed plants, whereas the interaction of BR and ABA regulates the expression of different genes to enhance stress tolerance [52, 55–58]. Furthermore, ABA insensitive (*abi1, abi2*) and BR perception (*bri1*-*1*) mutants reflect the interaction between ABA and BR. This interaction regulates various genes that play a key role in plant developmental process [GPA (G protein α-subunit1), involved in seed germination; DWF1 (DWARF1) and CPD (CONSTITUTIVE PHOTOMORPHOGENESIS AND DWARFISM), involved BR-biosynthesis] and stress responses (*SOD, POD, CAT, GR*, involved plant defense system) [26, 56, 57].

Ethylene is a plant hormone produced in all plant organs that plays a fundamental role in plant growth and development. Ethylene enhances plant tolerance to a wide range of biotic and abiotic stresses. A previous study reported that BR play a key role in biosynthesis of ethylene in plants [55]. Methionine, the starting point of the ethylene biosynthesis pathway, is converted into SAM (*S*-adenosylmethionine) with the help of methionine adenosyltransferases. SAM is first converted into ACC (1-aminocyclopropane-1-carboxylic acid) with the help of ACC synthase and then it is converted into ethylene regulated by enrichment of the BR signal pathway [3]. Another study suggested that BR significantly increased the transcription level of ethylene signaling biosynthesis genes including ripening related 1-aminocyclopropane-1-carboxylic acid (*ACC*), 1-aminocyclopropane-1-carboxylate synthesis1 (*ACS1*), 1-aminocyclopropane-1-carboxylate synthesis2 (*ACS2*), 1-aminocyclopropane-1-carboxylate synthesis3 (*ACS3*), (1-aminocyclopropane-1-carboxylate oxidase1) (*ACO1*), (1-aminocyclopropane-1-carboxylate oxidase1) (*ACO2*), and alternative oxidase (*AOX*) in cucumber under stress conditions [15]. Ethylene accumulation and biosynthesis are induced by BR treatment in tomato [55]. These results suggested to crosstalk between BR and ethylene in response to stress tolerance.

Cytokinin promotes cell division in roots and shoots [56, 57]. In wheat seedling, exogenous BR decline the enzyme activity and encoded gene expression of cytokinin oxidase contributes significantly to increase cytokinin level, indicate the involvement of BR in the regulation of cytokinin [59]. Overexpression of *BRI1* in *PYK10:CKX3* increased root and leaf growth, compared with wild type and similar to a CYTOKININ DEHYDROGENASE/OXIDASE 3 (*CKX3*) overexpression line in tobacco, are indicating the active crosstalk between cytokinin and BR, which both play important roles in several aspects of plant growth and development [58, 60, 61]. Another study showed that exogenous BR enhanced endogenous levels of SA, JA, and ethylene and concluded that crosstalk occurred between BR and other plant hormones in the signaling pathway under BR-induced stress tolerance [25]. Exogenous BR can also positively increase endogenous hormone quantity [62]. Our study (in press) shows that exogenous BR regulates ABA, IAA, ZR, and BR accumulation, but decreases GA4, JA and iPA accumulation in response to low temperature stress in cucumber. Therefore, the BR signal transduction pathways are involved in many transcriptional activities, signal transduction, and metabolic activities, which lead to significant resistance to a variety of stresses and protect plants from injury.

### Fruits and yield

Fruit development and yield have been intensively studied over the past decade and a key challenge for scientists is to improve yield per unit area. Increasing yield and promoting yield components are a key task for researchers to meet food demand, because the global population is increasing. However, biotic and abiotic stresses are a major barrier to meeting food demand because they significantly reduce plant growth, developmental processes, and yield [47, 63]. Earlier studies showed that BR significantly increase yield and yield components of mustard plant [6]. Foliar application and seed treatments of BR significantly enhanced growth of tomato, as well as number of fruits and weight per plant; the yield of pear and yellow passion fruit are also improved by BR application [64, 65]. Previous studies reported that BR played a positive role in fruit ripening and fruit growth of mango, and in the quality of pitaya [64, 66]. Thus, BR reduce the harmful effect of stress by activating a plant defense system (antioxidants) against stress conditions and leading to significantly increased growth, yield, and yield components in cucumber.

## Involvement of BR in tolerance to major stresses

Brassinosteroids is a plant steroidal hormone that plays an important role in a variety of plant physiological processes and adaptation to different kinds to abiotic and biotic stresses [15, 48]. Many studies suggest that BR regulate defense enzymes and hormones during the induction of plant stress responses. Here, we discuss some physiological and molecular mechanisms of the BR response to various stresses.

### Response to low temperature/chilling stress

Chilling stress is a major abiotic stress limiting plant growth and development in many parts of the world, strongly damaging plant physiological processes [63]. Low temperature leads to: arrested plant growth, disordered photosynthetic processes, negative effect on chlorophyll contents, and flower bud abortion, resulting in significant yield and economic losses. Exogenous BR application was shown to significantly increase resistance against low temperature stress in pepper [67], by modulating morphological, physiological, and biochemical characteristics of tomato [17], cucumber [15], and pepper [68] (Table 1). The authors of these studies concluded that BR alleviate the negative effect of low temperature and chilling stress by increasing chlorophyll contents, maintaining photosynthetic activities and carbohydrate metabolism, inducing changes in defense enzymes, reducing toxic ion contents, activating gene expression, increasing contents of endogenous plant hormones, and activating signal transduction pathways (Figs. 2, 3) [47, 69]. Pepper seedlings increased tolerance to chilling stress when treated with BR, as revealed by transcriptome analysis that BR upregulated the expression of thousands of genes in pepper under chilling stress (Fig. 4) [2]. Furthermore, the authors reported that BR increased endogenous salicylic acid and jasmonic acid and enhanced the ethylene biosynthesis pathway, suggesting that BR function via synergistic crosstalk with salicylic acid, jasmonic acid, and the ethylene signaling pathway to respond to chilling stress [15, 48]. BR activate the expression of cellular redox homeostasis-related genes (*GSTX1; probable glutathione S*-*transferase, PER72; peroxidase 72* and *CAT2; catalase isozyme 2*) to reduce the harmful effect of chilling stress [2]. These lines of evidence suggest that BR play a role against chilling/cold stress by activating cold stress response genes (in *Brassica* and *Arabidopsis*), signal transduction pathways (BR and ethylene signaling pathway), transcriptional levels of stress response genes (*SOD, POD, CAT, GR*), and defense systems (Fig. 2) [2, 17].

**Table 1:** BR response to various kinds of cold stresses

Stress	Crop	Year	Remarks	References
Low temperature	*Leymus chinensis*	2016	EBR enhance growth and development by improvement of biosynthesis of photosynthetic pigment and antioxidant enzymes activities under low temperature	[94]
Chilling stress	Pepper	2015	EBR play protective role against chilling stress by increasing photosynthetic capacity and antioxidant enzymes activates	[47]
Chilling stress	Grapevines	2013	Exogenous EBR antioxidant defense system, reduce oxidative damage and lipid oxidation against chilling stress	[63]
Low temperature	Tomato	2016	EBR significantly increase plant growth and developmental process and reduce low temperature effect	[17]
Chilling stress	Cucumber	2015	EBR involved ethylene biosynthesis to regulate plant defense system, to resist abiotic (chilling) stress	[15]
Chilling stress	Pepper	2016	EBR regulate thousand genes in response to chilling stress	[2]
Low temperature	Cucumber	2018	EBR play a key role to improve growth, endogenous hormones and antioxidant enzymes regulation under chilling stress	[95]
Chilling stress	Cucumber	2015	EBR showed a significant resistance to chilling stress and increase antioxidant enzymes capacity	[15]
Chilling stress	Pepper	2015	EBR promote photosynthetic capacity, carbohydrate metabolism and reduce stress effect	[68]
Temperature stress	Brassica	2013	EBR significantly reduces the negative effect and protect cell membrane by increase proline contents and antioxidant enzyme activity to enhance stress resistance	[96]
Freezing stress	Arabidopsis	2016	EBR control basic helix-loop-helix transcriptional factor that regulate cold stress resistance genes	[69]

### High temperature or heat stress

Plants have evolved pleiotropic and intricate regulatory functions to defend against biotic and abiotic stresses [37, 70]. During stress conditions, phytohormones (BR, SA, JA, ABA, and GA) play a fundamental role in signal transduction pathways and stimulate defense mechanisms of plants [51] (Table 2). Of these phytohormones, BR are reported to regulate plant growth and a broad spectrum of physiological responses to abiotic stresses (such as high temperature) [25]. BR showed a significant response to high temperature stress in banana [71], *Ficus concinna* [72], *Brassica*, and *Arabidopsis* [70], by maintaining physiological and antioxidant defense systems. *Ficus concinna* seedlings treated with BR and exposed to high temperature (28, 35, 40 °C) stress for 48 h resulted in significant increments in reduced glutathione (GSH), oxidized glutathione (GSSG), GSH/GSSG, ascorbate (AsA), oxidized ascorbate (DHA) contents, and increased antioxidant enzymes activity (SOD, POD, CAT, GR, APX) [72]. It can be concluded that BR could alleviate high temperature stress by increasing enzymatic and non-enzymatic antioxidant defenses and the glyoxalase system.

**Table 2:** BR response to various kinds of high temperature stresses

Stress	Crop	Year	Remarks	References
High temperature	Brassica	2013	Decreased lipid peroxidation, increase proline contents, protect cell membrane by EBR	[96]
Heat stress	Tomato	2005	EBR increase basic thermotolerance of germinating pollen	[97]
Heat stress	Rice	2016	BER increase pollen fertility, germination rate and reduce the effect of heat stress	[98]
Heat stress	Brassica	2002	EBR increase accumulation and biosynthesis of heat shock protein (HSPs), protect plant form heat stress	[99]
Lethal heat stress	Tomato/Brassica	1999	EBR induce expression of HSPs and significant increase plant survival under heat stress condition	[100]
High temperature	Rice	2015	EBR significantly enhance photosynthetic activity, chlorophyll, yield and yield components under stress	[65]
Heat drought	Wheat	2017	EBR increase significantly photosynthetic capacity by increasing RCA subunit and Rubisco activity under drought and heat stress	[45]
Thermo-tolerance	Tomato	2011	EBR significantly increase photosynthesis, chlorophyll, pollen germination % and bursting % yield and related parameters under heat stress	[101]
Heat stress	Tomato	2016	EBR enhance antioxidant enzyme activity, photosynthesis, chlorophyll fluorescence, electron transport rate and Rubisco activity under heat stress condition	[100]
Heat stress	Banana	2004	EBR significantly increase shoot length, shoot induction %, fresh weight and reduce injury % under heat stress in In vitro condition	[71]
High temperature stress	*Leymus chinensis*	2016	EBR enhance plant growth, antioxidant enzyme capacity, chlorophyll, GR, under high temperature stress	[102]

### Water deficit or drought stress

Water is crucial for growth and development of plants but may or may not be available in many parts of the world. Water defect or drought stress, are serious factors limiting the productivity of a variety of agricultural crops and can seriously damage antioxidant systems of plants, decrease chlorophyll contents, negatively affect photosynthetic activity, and also damage membrane stability [15]. Gram (*Cicer arietinum*) plants exposed to water stress and treated with BR had significant increases in fresh and dry weight, number of tillers, stem thickness, root activity, and nitrate reductase activity [73]. Radish seedlings treated with BR were exposed to water deficit stress [28], and results showed that BR increased the activity of plant antioxidant enzymes and reduced drought stress effects on plants. For sorghum [42], maize [74], *Robinia* [75], and tomato [62, 76], BR enhanced chlorophyll accumulation, amylase activity, total protein contents, stomatal conductance, photosynthesis, and membrane stability. Taken together, these findings suggest that BR not only alleviated the antagonistic effect of drought stress but also enhanced plant growth and yield.

### Saline or NaCl stress

Saline soil has a high concentration of soluble salts and an ECe of 4 dS/m or more. Salinity is one of the most serious factors limiting the productivity of agricultural crops. High salinity affects plants in several ways: alteration of metabolic processes; membrane disorders; irregular cell division and expansion; decreased photosynthetic activity and protein synthesis; increased ion toxicity; and enzymatic disorders. BR were reported to increase resistance of many plants to saline stress, including pepper [11], rice [24], canola [12], and *Brassica juncea* [40], as shown in Table 3. These findings showed that BR significantly increased plant tolerance to saline stress. Recent studies reported that BR increased physiological mechanisms against salt stress [11–13, 25]. The exogenous BR increased chlorophyll contents, photosynthesis parameters, and the activities of antioxidant enzymes, and reduced reactive oxygen species (ROS) and MDA contents to mitigating the harmful effects of salt stress [25]. Furthermore, BR showed significantly increased levels of endogenous hormones, decreased ion toxicity, and increased total amino acid contents under salt stress [22, 32, 77]. BR-treated cucumber seedlings exposed to polyethylene glycol (PEG) and cold stress significantly increased physiological parameters (height, root and shoot growth, leaf area and seedling index) and defense system enzymes [SOD, POD, CAT, APX, GR (glutathione reductase)], and reduced the contents of ROS (H_2_O_2_, O_2_^∙−^) and MDA. BR also increased transcription levels of ethylene signaling biosynthesis genes (*CsACS1, CsACS2, CsACS3, CsACO1, CsACO2* and *CsAOX*) in cucumber seedlings [48, 55]. The same results were reported in canola treated with BR under salt stress [13, 29, 32]. In summary, BR positively increase tolerance of plants in response to saline stress [78, 79].

**Table 3:** BR response to various kinds of salt and saline stresses

Stress	Crop	Year	Remarks	References
Salt	Strawberry	2011	EBR keep balance in plant nutrients, enhance plant antioxidant system, stabilize membrane stability and chlorophyll	[103]
Salinity	Mung bean	2010	Increase plant growth parameters, m membrane stability index, relative water contents, NR, CA activities, EC, H_2_O_2_ and chlorophyll SPAD by EBR treatment under stress conditions	[104]
NaCl/CdCl_2_	Bean	2011	EBR significantly enhance growth, yield by activating antioxidant enzymes under stress condition	[105]
Salt	Peppermint	2016	EBR play a protective role to alleviate salt stress effect and improve growth	[106]
Salt	Lettuce	2012	EBR have ability to enhance growth, GS enzyme activity, macro and micro nutrients uptake, improve cell membrane stability under salt stress	[107]
Salinity	Potato	2016	EBR significantly enhance in vitro potato adventitious root growth, root length, root number, bio mass, root activity, maintaining K^+^/Na^+^ homeostasis and antioxidant capacity	[108]
Salt	Eggplant	2012	EBR increase superoxide dismutase, guaiacol peroxidase, catalase and ascorbate peroxidase, and also increase essential ion contents under salt stress	[109]
Salt/copper	Cucumber	2013	EBR enhanced the level of antioxidant system (superoxide dismutase, catalase, peroxidase and proline) and improved growth parameters, both under stress and stress-free conditions	[110]
NaCl	Cucumber	2011	EBR reduce NaCl stress effect and increase germination, ACS and ACO genes expression (ethylene bio synthesis), ACO activity	[111]
Salinity	*Brassica juncea*	2017	EBR protect plant from salinity stress and enhance nitrogen, proline and ABA metabolism	[112]
Salt	Tomato	2016	EBR increase growth, proteins contents and antioxidant enzymes activities in in vitro growing potato.	[113]
NaCl	Ryegrass	2017	EBR enhance growth, Na^+^, root activity, protein contents, proline contents and antioxidant enzymes capacity	[25]
Saline	Pigeon pea	2013	EBR increase NR activity, amino acid, proteins contents, nutrients contents and increase defense system	[114]
Saline	Pepper	2013	EBR enhance growth and related parameters, reduce toxic ion effect	[11]
Salt	Rice	2012	EBR increase pea growth, activate different enzymes, chlorophyll, photosynthesis, proline and yield under salt stress condition	[115]
Salinity	Rice	2004	EBR significantly increase germination %, shoot and root length, fresh and dry weight, yield and yield components under salinity stress	[27]

### Heavy metal stress

Heavy metals are natural components of the earth’s crust and cannot be degraded or destroyed. Plants have a remarkable ability to uptake and accumulate heavy metals. High concentrations of all metals, including those that are essential or non-essential for plant growth and metabolism, cause toxic effects on metabolic pathways in plants [80]. The toxicity of heavy metals can have the following effects: blocking functional groups of important molecules (e.g. enzymes, polynucleotides); blocking the plant transport system for essential nutrients and ions; displacing essential ions from cellular sites; and inactivating or causing an imbalance of antioxidant enzymes. BR play a pivotal role in overcoming heavy metal toxicity and improving plant growth [80–85] (Table 4). In *Helianthus tuberosus* plants exposed to cadmium [86], Cd significantly reduced plant growth, chlorophyll contents, net photosynthetic rate, and enzymatic activities (of carbonic anhydrase and nitrate reductase). However, BR significantly reduced the toxic effect of Cd in *H. tuberosus* and enhanced plant height, chlorophyll contents, photosynthetic rate, and enzymatic activities [80, 87]. BR-treated plants had lower bioaccumulation of heavy metals than untreated plants in *Brassica juncea* [80, 83–85].

**Table 4:** BR response to various kinds of heavy metal stresses

Stress	Crops	Year	Remarks	References
Aluminum	Mung bean	2008	EBR significantly increased plant growth, chlorophyll under Al stress condition	[85]
Iron deficiency	Cucumber	2012	EBR regulate cucumber growth, stimulate ferric reductase activity (FRO), FRO1 and IRT1 (Fe transporters) genes expression and reduce Fe translocation from roots to shoots, reduce Fe deficiency effect	[116]
Heavy metal	*Chlorella vulgaris*	2000	EBR significantly overcome the inhibitory effect of heavy metals and significantly regulate plant growth	[87]
Heavy metal	*Chlorella vulgaris*	2010	EBR significantly reduce heavy metal stress by activation antioxidant enzymes activities	[80]
Cadmium	*H. tuberosus*	2012	EBR restore photosynthetic capacity and reduce Cd effect	[81]
Cadmium	Winter rape	2005	EBR enhanced antioxidant enzymes capacity to reduce Cd effect and significantly improve photosynthesis efficiency in rape	[117]
Copper	*Brassica juncea.* L	2007	Pre-sowing treatment of EBR enhance seedling by activating antioxidant enzymes capacity, reduce metal uptake, increase protein biosynthesis	[118]
Chromium	Chickpea	2008	EBR significantly alleviate chromium effect, increase chlorophyll fluorescence, photosynthesis and antioxidant enzymes capacity and ultimately increase tobacco seedling growth	[89]
Copper	Radish	2010	EBR improved radical scavenging activities, increase antioxidant enzymes capacity, increase ascorbic acid, phenols and proline contents, because of it plant growth were improved significantly	[119]
Heavy metal	Maize	2013	EBR have a stimulating effect on seed germination and seedling growth. It reduce heavy metal induce electrolyte leakage form maize cells	[120]
Nickel	*Brassica juncea*	2007	EBR significantly enhanced *Brassica juncea* growth, chlorophyll contents, photosynthetic capacity, Nitrate reductase and carbonic anhydrase activity and activate defense system	[121]
Nickel	Wheat	2010	EBR enhance wheat seedling growth and related parameters, photosynthesis, chlorophyll, antioxidant enzymes capacity and activate enzymes	[122]
Lead	Fenugreek	2014	EBR improve plant growth, biomass, photosynthesis pigment, photosynthesis and reduce Pb toxic effect	[123]
Cadmium	Tomato	2011	EBR play a protective role against Cadmium stress by increasing plant defense system and activating enzymes	[46]

BR alleviate the harmful impact of heavy metal stress on growth by preventing and increasing chlorophyll contents and photosynthetic activity and increasing synthesis of phytochelatins. BR also reduce the toxic effect of cadmium and copper on rape and mustard plants [6], maintaining efficient photosynthetic activities and lessening the damage to reaction centers [81]. Similarly, a significant reduction of heavy metal absorption in plants has been reported. For example, around 50% lower lead was reported in beetroot treated with BR compared to control [88] and BR significantly improved the performance of mustard, mungbean, and chickpea under cadmium, aluminium, and nickel stresses [4, 88–90]. Activities of the enzymes CAT, POD, carbonic anhydrase, and NR are also enhanced by BR under nickel stress in mungbean [6, 83, 85]. BR enhance the enzyme activities of antioxidants [CAT, POD, SOD, GR (glutathione reductase)], increase proline contents, and reduce reactive oxygen species (ROS) and malondialdehyde (MDA) accumulation [80, 81]. A similar defense pattern for foliar application of BR was reported: plant growth, photosynthesis, antioxidant enzymatic activities, and proline contents were significantly increased in aluminium-stressed mungbean plant [85]. BR improved plant growth and developmental processes under stress from different heavy metals by activating antioxidant enzymes and expression of defense genes [*cAPX, CAT, MDAR* (monodehydroascorbate reductase) and *GR*]. Recently four new genes were identified: *Cs594* (Aphloem protein 2, is known as phloem defense-related protein)*, Cs623* (an MLP-like protein; is also known as phloem defense-related protein)*, Cs453* (a member of Auxin/indole-3-acetic acid protein family, encoded as IAA14)*, Cs579* (functions as a molecular chaperone). These genes function as defense activators in BR-treated cucumber [48], and increase: proline contents [11, 12], gene expression of endogenous plant hormone biosynthesis, photosynthetic machinery protection, and expression of related genes [2, 3, 22, 81]. Overall, BR significantly alleviate the harmful effects of heavy metals and improve plant growth.

### Biotic stress

In field conditions, plants face different kinds of stress conditions, both biotic (damage by any living organism: bacteria, viruses, fungi, parasites, beneficial and harmful insects) and abiotic (environmental factors such as low and high temperature stress, salinity etc.). Like animals, plants also have an immune system, which provides resistance to external stressors (biotic and abiotic stress). To effectively combat invasion by infectious pathogens and herbivorous pests, plants make use of preexisting physical and chemical barriers, as well as inducible defense mechanisms, which become activated upon attack; plant defenses function as a unit to reduce harmful effects of biotic stresses. The induced defense system of plants against biotic stresses is similar to defense against abiotic stress. For both types of stress, the induced defense system is regulated by complex interconnected signal transduction pathways in which plant hormones (ABA, ETH, JA, SA and BR) play a fundamental role [25, 51, 53]. Application of BR at low concentrations significantly improves growth and yield and increases resistance to viral and fungal pathogens in cucumber, tobacco, and tomato [43, 91]. However, the levels of protection and effectiveness depend upon method and time of BR application. In a recent study of the fungal disease cedar-apple rust [92], the flavonoid compounds anthocyanin and catechin, and transcript levels of induced *MYB* genes (*MYB30*) were increased in rust infected tissues. In another study, *MYB30* genes directly regulated *BES1* in *Arabidopsis*. *BES1* is a key gene of the BR signal transduction pathway, and *AtMYB30* mutants and *BES1* interact with each other and promote BR targeted genes both in vitro and in vivo [60]. Furthermore, plant hormones ABA, ETH, JA and SA were at the highest level in rust infected apple plants [92], whereas in pepper plant treated with BR and exposed to chilling stress, contents of plant hormones SA, JA, and ETH were significantly increased [2]. These results suggest that BR functions via synergistic crosstalk with SA, JA, and ETH signaling pathways to respond to chilling stress. These findings show that BR play an essential role in biotic stress tolerance by activating enzymes, biotic resistance genes, antioxidants, hormones, transcriptional factors, and signaling pathways to reduce biotic stress damage.

## Concluding remarks

Recently, intensive research has focused on understanding the mechanisms of BR, signaling transduction pathways, and BZR1/BES1 transcription factors in responding to stress conditions. Based on this review, we conclude that BR enhance plant tolerance to biotic and abiotic stresses, through a complex pathway to regulate the plant defense system, by activating BZR1/BES1 transcription factors. BR regulate ROS production in plants under stress, and unbalancing of ROS scavenging leads to oxidative bursts, which have adverse effects on plants. BR activates CBFs (C-repeat binding factors), key transcriptional factors, which are responsible for controlling the regulation of genes that respond to low temperature stress. Similarly, transcriptomic analysis discovered that BR regulate thousands (39,829) of genes under stress conditions to enhance the plant defense system.

In this review we discussed various aspects of BR and regulatory mechanisms in stress conditions and used key points from different studies to clarify how BR increase stress tolerance. While BR regulate thousands of genes, it is not clear which other transcription factors and signaling proteins interact with *BZR1* and *BES1* to enhance stress tolerance. It will be interesting to explore the role of the BR signaling pathway, hormone interactions, and crosstalk at organ, tissue, and cell levels to better understand how plants respond to environmental stresses. Several studies showed the potential of BR in stress resistance, but minimal attention has been given to studying their interactions with ion homeostasis and transporters (N, P, K, Ca, Mg etc.) for identification and functional characterization of various nutrient transporters. Future research will offer the possibility for genetic engineering of crop varieties and improving the acquisition of nutrients to reduce the amount of fertilizer application. Moreover, very little attention has been given to explore the role of BR in yield. Therefore, future studies are needed to understand detailed mechanism, and explore the role of BR in growth and development in responding to environmental cues.

## Abbreviations

ABA: abscisic acid
BZR1: Brassinazole resistance 1
BES1: BRI1-EMS suppressor 1
CAT: catalase
ETH: ethylene
GA: gibberellin
GOGAT: glutamate synthase
GR: glutathione reductase
GSH: glutathione
GSSG: oxidized glutathione
H_2_O_2_: hydrogen peroxide
JA: jasmonic acid
MDA: malondialdehyde
NR: nitrate reductase
POD: peroxide
ROS: reactive oxygen species
SA: salicylic acid
SOD: superoxide dismutase
ZR: zeatin riboside
